# Enantioselective Box Behenken Optimized HPLC-DAD Method for the Simultaneous Estimation of Alogliptin Enantiomorphs in Pharmaceutical Formulations and their Pharmacokinetic Study in Rat Plasma

**DOI:** 10.15171/apb.2019.018

**Published:** 2019-02-21

**Authors:** Ravi Kant, Ramesh Babu Bodla, Rubina Bhutani, Garima Kapoor

**Affiliations:** Pharmaceutical Chemistry Department, Delhi Institute of Pharmaceutical Sciences & Research, University of Delhi, Sector 3 Pushp Vihar, Mehrauli Badarpur Road, New Delhi -110017, India.

**Keywords:** Alogliptin enantiomers, Box–Behnken design, Pharmacokinetics, HPLC-DAD, SPE

## Abstract

***Purpose:*** A stereoselective high performance liquid chromatographic analytical method with photodiode array detector was developed and validated as per the International Conference on Harmonization (ICH) guidelines for the determination of alogliptin (ALO) enantiomers in formulations and rat plasma.

***Methods:*** Enantiomeric separation was performed on a Phenomenex Lux Cellulose-2 chiral column. Box-Behnken design was used to identify the optimum conditions of the three independent variables for the desired output responses.

***Results:*** The HPLC peaks of ALO enantiomers and the internal standard pioglitazone were achieved before 8 min with a resolution of 0.77 min between R and S enantiomer and resolution of more than 2.0 between each enantiomer and pioglitazone (internal) with more than 95% recovery. The linearity range and the limit of quantification of both the enantiomers in rat plasma were 10-70 ng mL^-1^ and 1.2 ng mL^-1^ respectively.

***Conclusion:*** The developed method after validation was successfully applied for estimation of ALO enantiomers in formulations. Single oral dose of 25 mg of the ALO racemate tablets were administered to a group of 6 healthy rats for a comparative pharmacokinetic study of both the enantiomers.

## Introduction

**Figure 1:**
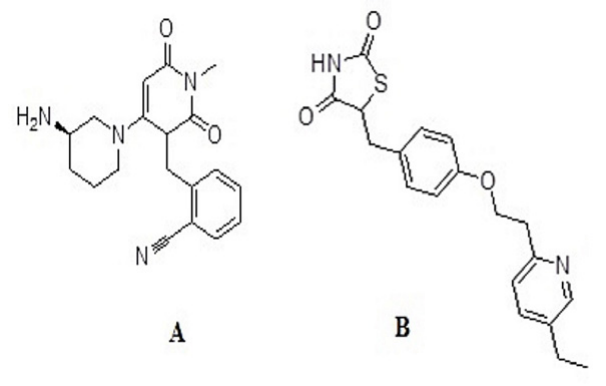
Chemical structure of (A) Alogliptin and (B) Pioglitazone.

Type 2 diabetes mellitus is a life-threatening metabolic disorder characterized by high levels of glucose levels due to impaired insulin secretion or insulin resistance or both.^1,2^ Alogliptin (ALO) with IUPAC name as 2-({6-[(3*R*)-3-aminopiperidin-1-yl]-3-methyl-2,4-dioxo-3,4-dihydropyrimidin-1(2*H*)-yl}methyl) benzonitrile (Figure 1) is a selective orally administered antidiabetic drug that belongs to dipeptidyl-peptidase-4 (DPP-4) inhibitor class.^3^ ALO improves the glycemic control in type 2 diabetes mellitus patients. Its mechanism of action consists of inhibiting the degrading enzyme dipeptidyl-peptidase-4 and thus increasing the endogenous glucagon-like peptide-1, an incretin hormone, and gastric inhibitory polypeptide hormone. The glucagon-like peptide-1 has a greater stimulatory effect on insulin secretion than that of blood glucose.^4^ The pharmacokinetic and pharmacodynamic profiles of ALO supports the use of a once-daily dosing regimen. ALO is well tolerated, with no dose-related toxicity.^5-7^ As per the International Conference on Harmonization (ICH) guidelines, the enantiomeric nature and quantity and the quantitative assay tests for chirality are required for product specifications.^8^ Chromatographic techniques, most commonly HPLC have been given preference for the separation of enantiomers for the past many decades.^9-12^ Chiral HPLC provides quick and reliable methods for chiral separation and permits on-line detection and quantitation of both mass and optical rotation of chiral forms using suitable detection devices.^13,14^ US FDA and other drug regulatory agencies have made it compulsory for the manufacturers to explore each enantiomer of the chiral drug separately.^15^ ALO exists mostly as the (R)-enantiomer (R-ALO) and is subjected to a small or no chiral conversion to the (S)-enantiomer (S-ALO) in vivo. The R-ALO is the active moiety and is >150-fold more active against DPP-4 than the S-ALO.^16^

As per the US-FDA it is desirable to develop analytical methods for the stereoisomeric drug entities in the early phase of drug development.as they have important pharmacological and pharmacokinetic effects on humans as well as animal body.^17^ The traditional approach of method development has been to sequentially change one variable at a time until a suitable method is developed, also called the one-factor-at-a-time approach. The problem with this approach is that many experiments are needed making it a tedious process and still they frequently fail to predict the most optimum condition.^18^ This approach is unable to detect the possible interaction between the factors leading to misinterpretation of results.^19^ Now the new approach in analytical method development is based on the use of design of experiments (DOE) which has recently become quite popular.^20^ The major factors affecting the performance of the analytical methods can be better understood.^21^ The detailed understanding of the probable risks and the associated interactions among the method variables is possible on the basis of the principles of DOE.^22^ The DOE approach involves scrutinizing and optimizing the method using experimental designs.^23^ Statistical DOE method is a quality by design approach which helps to develop a design space. A design space is an experimental region where small changes in method parameters will not significantly affect the results of the method thus strengthening its robustness.^24,25^

A large number of methods have been developed using the DOE approach either single or in fixed dose combination formulations both in bulk drugs as well as in pharmaceutical dosage forms.^26-29^ Central composite design method, Doehlert and Box-Behnken design methods are the three most commonly used statistical response design methods. Of these, the Box-Behnken method (BBD) has been proved to be less expensive due to lowest number of runs and experimentally more convenient than the other experimental designs (Central Composite Design, Doehlert, Factorial Designs etc). It is a class of rotatable or nearly rotatable second order design which does not allow combinations in which all the factors are concurrently at their highest or lowest levels thus avoiding extreme conditions for which unsatisfactory results might occur.^30,31^

The review of literature revealed that a number of achiral and chiral chromatographic methods have been published for the analysis of Alogliptin in bulk drugs, pharmaceutical formulations as well as in plasma.^32-36^ Also, many chromatographic as well spectrophotometric methods have been published using the DOE methodology; especially Box-Behnken method for the estimation of drugs in single as well as combined dosage forms.^37-43^ But till date, there have been no published reports about the estimation of ALO enantiomers by HPLC using the DOE optimization method in plasma.

In this paper, we have tried to develop a simple, specific and accurate chiral HPLC method using Box-Behnken design optimization method for the quantitative estimation of R and S-ALO in plasma and its application to enantioselective pharmacokinetics study in rats. Selection of the optimum experimental conditions in terms of mobile phase composition, pH and flow rate was made by using Derringer’s desirability function.

## Materials and Methods

### Materials and reagents

Pure compounds of racemic ALO, pioglitazone (PIO), R-ALO and S-ALO (Figure 1) were received as gift sample from the Sun Pharmaceutical Industries Limited (Gurugram, Haryana, India) containing 99.82%, 99.59%, 99.39% and 99.65%, respectively as per the certificate of analysis obtained. HPLC grade methanol and formic acid was purchased from Merck (Darmstadt, Germany). ALO tablets (Perrigo Company plc, Dublin Ireland) of 25 mg strength were bought from a retail chemist shop. The C-18 solid phase extraction cartridges (150 mg, 6 mL) were purchased from Waters (India) Private Limited (DLF Tower-B, Jasola, New Delhi). Blood of 6 healthy rats from retroorbital plexus (behind the eyes) venous sinus bleed were collected and centrifuged. The pooled supernatant plasma (control) was then stored at -20°C for further use.

### Chromatographic apparatus and conditions

The HPLC system (Shimadzu Corporation, Prominence Modular UFLC, Kyoto, Japan) contained binary pump LC 20AD, System Controller CBM-20A and a Photo-diode Array detector SPD-M20A. The chromatographic system is equipped with a 7725i injector with a 20 µL sample loop (Rheodyne, CA, USA). The chromatographic separations were performed on Phenomenex Lux Cellulose-2 chiral column (Cellulose tris [3,5-dimethylphenylcarbamate], 250 mm x 4.6 mm internal diameter, 5 μm particle size). The column was maintained at 25°C using oven throughout analysis. The LC Solutions software was used for acquiring data and processing.

Both the ALO enantiomers and the PIO were analyzed at the wavelength 230 nm within a run time of 10 minutes using a 20 µL loop (injection volume). Whatman membrane nylon filters (pore size 0.45 μm, diameter 47 mm) were used to filter the mobile phases and Chromafil Xtra Syringe Filters (PA-45/25, 0.45 µm, 25 mm diameter) to filter the samples.

### Method optimization

The optimization of chromatographic parameters was performed by using Box-Behnken design using the Design-Expert 11 software (Stat-Ease Inc., Minneapolis, USA). In the preliminary studies using one-factor-at-a-time approach different solvent types were tried and finally, methanol and 0.01% formic acid were selected on the basis of better peak shape and resolution between the peaks. Further optimization was done using Box-Behnken design. In this design methanol % (X_1_), flow rate (X_2_) and pH of the buffer (X_3_) were selected as the independent variables and R-ALO (Y_1_) and S-ALO (Y_2_) as dependent variables. The study of the individual and combined effects of three independent variables on the observed responses was performed using the Box-Behnken design. According to the Box-Behnken experimental design, 17 experimental trial runs were performed on the HPLC for the three factors and their effect on the investigated responses was observed. The coded levels of independent variables and their values are presented in Table 1. A total of 17 experimental runs were performed as per the Box-Behnken design (Table 2) in a randomized manner so as to minimize the effects of independent uncontrolled variables that may influence the results.

**Table 1:** Coded levels of variables and their values

**Variables**	**A (Low)**	**B (Medium)**	**C (High)**
Methanol (%)	40	55	70
pH of the buffer	3	3.5	4
Flow rate (mL/min)	0.8	1	1.2

**Table 2:** Box-Behnken design of three variables and the experimental observed responses

		**Factor 1**	**Factor 2**	**Factor 3**	**Response 1**	**Response 2**
**Std**	**Run**	**A: % Methanol**	**B: pH buffer**	**C: Flow rate (mL/min)**	**Retention time (R-isomer) (min)**	**Resolution between R & S isomer (min)**
1	1	40	3	1	7.263	1.588
8	2	70	3.5	1.2	6.647	0.904
4	3	70	4	1	6.843	1.112
3	4	40	4	1	7.259	1.494
13	5	55	3.5	1	7.042	1.326
15	6	55	3.5	1	7.026	1.308
11	7	55	3	1.2	6.874	1.118
2	8	70	3	1	6.793	1.002
16	9	55	3.5	1	7.073	1.306
9	10	55	3	0.8	7.209	1.611
5	11	40	3.5	0.8	7.348	1.611
17	12	55	3.5	1	7.021	1.313
12	13	55	4	1.2	6.921	1.313
10	14	55	4	0.8	7.211	1.414
6	15	70	3.5	0.8	6.994	1.147
7	16	40	3.5	1.2	7.113	1.405
14	17	55	3.5	1	7.056	1.343

Experimental data was evaluated following a quadratic second-order polynomial model:

Y = α_0_ + α_1_X_1_ + α_2_X_2_ + α_3_X_3_ + α_12_X_1_X_2_ + α_13_X_1_X_3_+ α_23_X_2_X_3_ + α_11_X_12_+ α_22_X_22_+ α_33_X_32_ + · · ·

­­­Where, Y is the measured response associated with each factor level combination; α represents the coefficients calculated by multiple regression analysis and X_1_, X_2_ and X_3_ are the effects of independent factors. The terms X_1_X_2,_ X_1_X_3_ and X_2_X_3_ represents the interaction terms between variables and X_11,_ X_22_, X_33_ represents the quadratic terms of independent variables.

Statistical evaluation of the responses was done using the analysis of variance (ANOVA) procedure to find the significant or insignificant differences in the responses obtained from the design matrix. The statistical significance of the fitted model was expressed by the F-test and the P value and its quality was checked by the coefficient of determination (R^2^). The optimum condition was selected using the derringer desirability function. Finally, on the basis of the numerical and graphical optimization procedure, the most optimum mobile phase composition was selected.

### Tablet assay

#### Primary stock and working solutions

Primary stock solutions of R-ALO and S-ALO (1 mg/mL) were prepared separately in methanol. Standard working solutions of R-ALO (40 μg/mL) and S-ALO (40 μg/mL) for preparing the calibration standards were made from stock solutions by further dilution.

#### Sample solutions of ALO tablets

Sample solutions of ALO tablets was made from a marketed brand (20 tablets) by calculating their total and then individual average weights and then pulverizing them to a powdered form. Then 25 mg equivalent of the mixed powdered tablets of ALO was blended with 25 mL methanol in a volumetric flask (25 mL) and filtered.

Now 400 μL of this solution was taken in a 10 mL volumetric flask and volume was make up with methanol thus making the final sample solution.

#### Plasma samples assay

#### Plasma stock and quality control (QC) samples

The stocks of racemic ALO (1 mg mL^-1^), R-ALO (1 mg mL^-1^), S-ALO (1 mg mL^-1^) and the internal standard PIO (1 mg mL^-1^) were prepared in methanol and stored at 4°C. The working solutions of both the enantiomers were prepared from the above stock solutions in the range of 20, 40, 60, 80, 100, 120 and 140 µg mL^-1^. Now 5 mL of each of the appropriate working plasma samples were spiked to 5 mL of control pool rat plasma thus making a concentration range of 10, 20, 30, 40, 50, 60 and 70 µg mL^-1^. Now by further dilution the standard curve solutions of both the enantiomers were prepared in the range of 1, 2, 3, 4, 5, 6 and 7 µg mL^-1^.

Five different concentration levels of R-ALO and S-ALO quality control samples in rat plasma were made at concentrations 1.5, 2.5, 4, 5.5 and 6.5 µg mL^-1^ and stored at -20°C. On each analysis day, one set of each of quality controls and standard samples were analyzed in the same way as plasma samples.

#### Dosing and sample collection

Two groups containing 6 rats each were made and after fasting overnight the R-ALO and S-ALO enantiomers were given orally at a single dose of 3 mg kg^-1^ to each separate groups. Blood was collected through rat retro-orbital plexus into heparinized tubes after 15, 30 min and 1, 1.5, 2, 3, 4, 6, 8 and 12 hours of drug administration. Samples were centrifuged for 10 minutes and plasma was separated and stored in refrigerator till further analysis.

#### Preparing the plasma samples

When the analysis has to be done, the stored plasma samples (at -20°C) are uniformly thawed on a mixing apparatus (vortex). The C-18 extraction cartridges (SPE) were washed with methanol (4x1 mL) and then same amount water. After that samples were passed through it and collected in conical glass tubes. The C-18 extraction cartridges are again washed with methanol (4x1 mL) followed with water (4x1 mL). Now the samples collected in the glass tubes are injected into the HPLC system through the manual injector fitted with 20 µL loop and analysis is performed.

#### Validation of the developed method

The developed methods were validated as per ICH^44^ and FDA^45^ Guidelines.

#### Specificity and the effect of the plasma matrix

The method specificity in the estimation of ALO in the presence of excipients in the tablet formulation was tested. The blank tablets (without active ingredient) containing only the excipients, the tablet formulation samples as well as the standards were tested using the same developed HPLC method and test results were compared. The chromatograms showed the specificity of the method in the presence of the excipients.

#### Standard curve (linearity)

Linearity was analyzed using seven different concentrations by further diluting the working standard solutions. Standard curves were plotted between peak areas versus concentration and regression analysis was performed. Each injection was made in triplicates. The acceptance criteria being that each of the standard concentration must be 100 ± 2% with relative standard deviation (RSD) lower than 2% and the correlation coefficient (r) of the regression line must be higher than 0.999.^44^ For the plasma samples, a calibration curve was made by plotting the peak area ratio of the ALO enantiomers and the internal standards versus the concentration of the individual enantiomers. Seven concentrations between 1 µg mL^-1^ to 7 µg mL^-1^ of ALO analytes in plasma matrix was used for establishing the linearity on which statistical analysis was performed.

The acceptance criteria for standard concentration was that they must be in the range 100±10% with RSD lower than 10%.^45^

#### Precision of the assay

The intra-day and inter-day precision and accuracy of the method for estimation of ALO analytes in tablet formulation were calculated in terms of % bias (recovery and %RSD).

Now 80%, 100% and 120% of the standard ALO samples of the actual amount expected in the real tablet sample were added in five repeats at each level. Then the analytes were evaluated for precision and accuracy in the same way as the tablets sample preparation method indicated above. Accuracy was determined as the ratio of calculated value to actual value, and precision in terms of the %RSD.

The acceptability rule involved accuracy within ±2% deviation from the actual amount and precision below RSD 2%.^44^ In case of rat plasma, the accuracy and precision were analyzed on the quality control (QC) samples in five repeats in a single (intraday) and three continuous (interday) days.

#### Robustness

The robustness of the method was executed by testing its capacity to withstand slight minor changes in the wavelength, mobile phase composition (i.e. methanol %) and the flow rate (1±0.2 mL min^-1^). For the robustness study the retention time of the peaks and the resolution between the analytes was analyzed.

#### Extraction recovery

Extraction Efficiency was determined at the LLOQ (10 ng mL^-1^) and at the five QC levels (15, 25, 40, 55 and 65 ng mL^-1^, n = 5). The calculations of the extraction recoveries were done by comparing the area under curve ratios of ALO analytes in the rat plasma samples added with ALO analytes before extraction with those plasma samples in which ALO analytes was spiked after extraction.

#### Stability studies

The studies on stability of ALO enantiomers was performed in plasma solutions from the quality control samples under different conditions at ﬁve concentration levels. The QC samples were subjected to (a) short-term or benchtop stability by thawing and then maintaining the frozen samples for 8 hours at room temperature before analysis, (b) freeze-thaw stability (3 cycles) by allowing the frozen samples stored at -20°C to thaw at room temperature followed by freezing at -20°C and again thawing, thus repeating the same process three times prior to analyzing in HPLC, (c) long-term stability, where analytes are stored at -20°C for 20 days followed by analysis in HPLC, and (d) post-preparative stability by storing the treated samples at 4°C for one day followed by analysis. The procedure for processing or treating the samples is expained in tablet and plasma assay section above. All of the analysis was done in 5 repeats.

#### Application to the pharmacokinetic study

A preclinical pharmacokinetic study of ALO enantiomers in the rats (six) was performed. The animals were housed in cages at room temperature (25± 2)°C, exposed to 12/12 hours each of light and dark cycle under relative humidity (60–70)% throughout the experimental period with easy availability of rat feed and water. Prior to drug administration the rats were fasted for 10 hours with easy availability of water. 500 μL of blood was taken from the rat retro orbital sinus plexus after single dose of 10 mg kg^-1^ ALO solutions at each time point of 0.25, 0.5, 1, 2, 4, 8, 12 and 24-hour. The samples were centrifuged at 4000 rpm for 10 minutes, plasma separated and stored at -20°C until analysis. After that 0.1 mL of plasma samples after spiking with internal standards were processed and analyzed for ALO concentrations using PK solver, a freely available menu-driven add-in program for Microsoft Excel.^46^ The pharmacokinetic parameters were obtained from the plasma concentration-time data. The peak plasma levels (C_max_) and the time taken to attain C_max_ (T_max_) were calculated using the concentration-time readings. Now 0.693/k_e_ is the elimination half-life (t_1/2_) of ALO where k_e_ (rate constant) was calculated from the concentration-time plot. The linear trapezoidal method was used to calculate the area under the plasma concentration-time curve from zero to the last measurable plasma concentration point (AUC_0–12_). Extrapolation to time inﬁnity (AUC_i_) was determined by the combination of AUC_t_ and AUC_i,_ where AUC_i,,_ is the residual area of drug from time t to inﬁnity and is the ratio of final drug concentration calculated in plasma and the rate constant k_c_. V_d_ is the volume of distribution and MRT_t_ is the mean residence time. C_L_ is the clearance rate and is equal the ratio of oral dose and AUC_i_.

## Results and Discussion

### Optimizing the analytical method

#### Optimization using Box-Behnken design method

**Figure 2:**
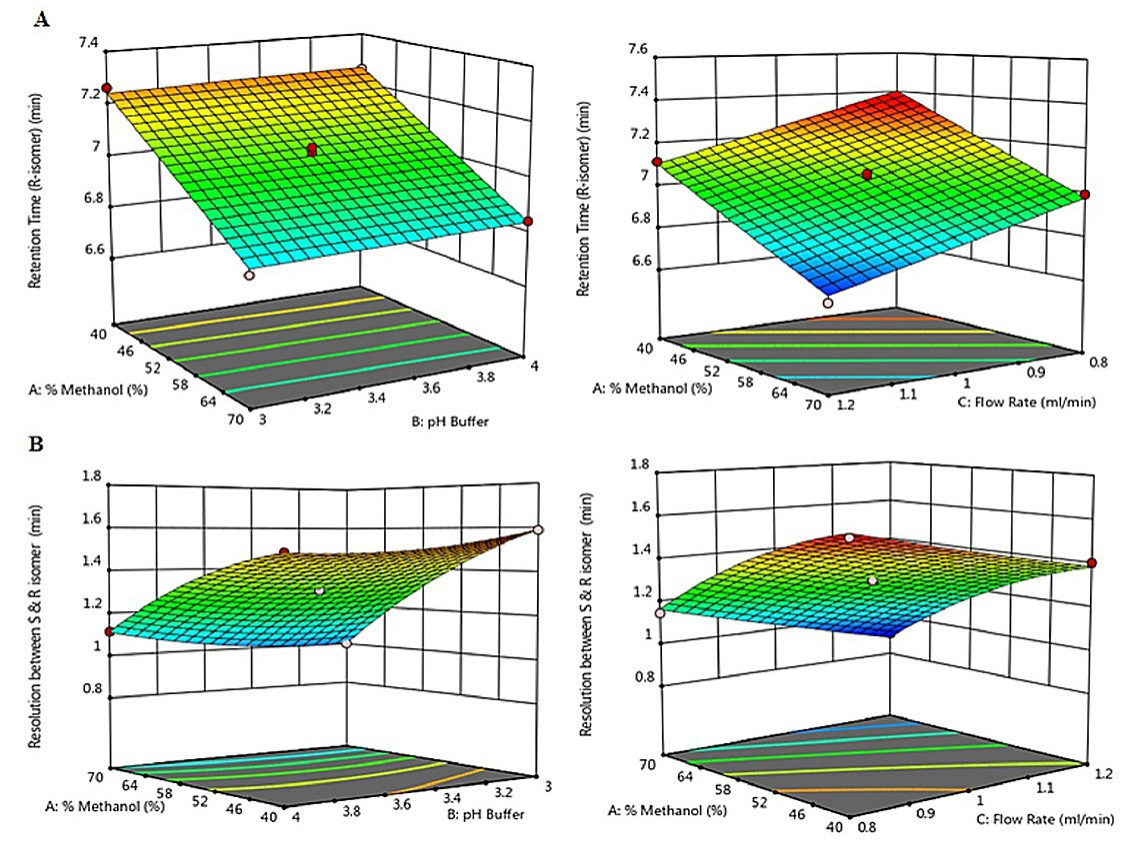
3D response Surface plots showing effects of %Methanol, pH of Buffer and flow rate on A. Retention Time of R-isomer and B. Resolution between R and S- isomer

Initially, trial and error method was used to select the suitable solvent system. Different solvents like hexane, isopropyl alcohol, acetonitrile, and methanol along with different buffers were tried. Finally, methanol and 0.01% formic acid were selected on the basis of better peak shape and satisfactory resolution between the peaks of enantiomers. Further optimization of the mobile phase composition was done by the BBD; an experimental design and statistical analysis method using Design Expert^®^ 11.0 software, Trial version. A total of seventeen tests were carried out in randomized order. The response surface methodology was used to understand the relationship between the dependent and the independent variables. The resulting graphs were shown in Figures 2A and 2B where one factor was kept constant at its center value.

An empirical second-order polynomial model was generated from the experimental results obtained from the proposed matrix whose coefficients were estimated by least square regression analysis.

Response variables, the retention time of R-isomer and the resolutions between the R and S isomers are markedly affected (*P* < 0.05) by the fluctuations of the independent variables methanol% v/v, pH of buffer and flow rate. The response variables, tailing factors and theoretical plates are not affected by the variations of the independent variables and are thus not included in the statistical model. Figure 2A and 2B shows the 3D response plots and depicts the relation between the three independent variables namely the methanol% v/v (X_1_), pH of the buffer (X_2_), and the flow rate (X_3_) on dependent variables, the retention time of R-isomer (Y_1_) and the resolution between the R and S isomer (Y_2_). From the 3D response plots Figure 2A and 2B, it can be observed that the response variable, the retention time of the R-isomer is dependent on the methanol concentration, flow rate and the pH of the buffer as the influential. A linear plot (Figure 2A) was obtained indicating a proportionate decrease in retention time of R-isomer with increasing concentration of methanol and flow rate, whereas a curvilinear plot (Figure 2B) obtained is indicating an increase in resolution between R and S isomer with a decrease in methanol and increase in flow rate. pH of the buffer has little effect on both the dependent variables.

The polynomial second degree model with the quadratic domain for response Y_1_ and with the linear domain for response Y_2_ with the highest regression value of 0.9844 and 0.9946 respectively was suggested by the design among the various models.

ANOVA was applied for the statistical evaluation of the fit of model to the data. There was not much difference between the calculated and the experimental models as shown by the P value of the lack of fit test. Also, the suitability of the BBD was established by the high regression values (>0.98) and low deviation values (standard deviation <0.03) (Table 3). From the mathematical polynomial equations created by ANOVA we can understand the relation between independent factors as well as the measured responses in a better way (see Table 3).

**Table 3:** Regression Model and Statistical Parameters obtained from ANOVA

**Response**	**R²**	**Adjusted R²**	**SD**	**CV %**	**Sum of squares**	**df**	**Mean square**	**F-value**	***P*** ** value**
Retention time (R-isomer): Y_1_	0.9844	0.9809	0.0258	0.3662	0.547	3	0.1823	274.3	< 0.0001
Regression model	8.49393-0.014217X_1_+0.02375X_2_ -0 .754375X_3_									
Resolution between R-isomer and S-isomer: Y_2_	0.9946	0.9878	0.0227	1.73	0.6722	9	0.0747	144.4	< 0.0001
Regression model	8.66927 – 0.008115X_1_ – 2,42920X_2_ -4.22604X_3_ +0.0068X_1_X_2_ -0.003083X_1_X_3_ +0.98X_2_ X_3_ -0.000261X_1_^2^+ 0.1541X_2_^2^ +0.156875X_3_^2^									

The magnitude of the coefficients of the regression equations can be used to assess the statistical significance and the relative effects of the interactions between the parameters. We can observe from these equations that the independent factors (X_1,_ X_2_ and X_3_) with positive sign have an increasing effect and those with negative signs have decreasing effects on the measured responses. In case of retention time Y_1_, the independent factors methanol% and the flow rate are negative while buffer pH was found to be positive. Similarly, for the resolution between R and S-isomer (Y_2_) all the three independent variables were negative. Desirability function was applied, so as to find the most optimal chromatographic conditions. It has values ranging between 0 being completely undesirable to 1 being fully desirable.

**Figure 3:**
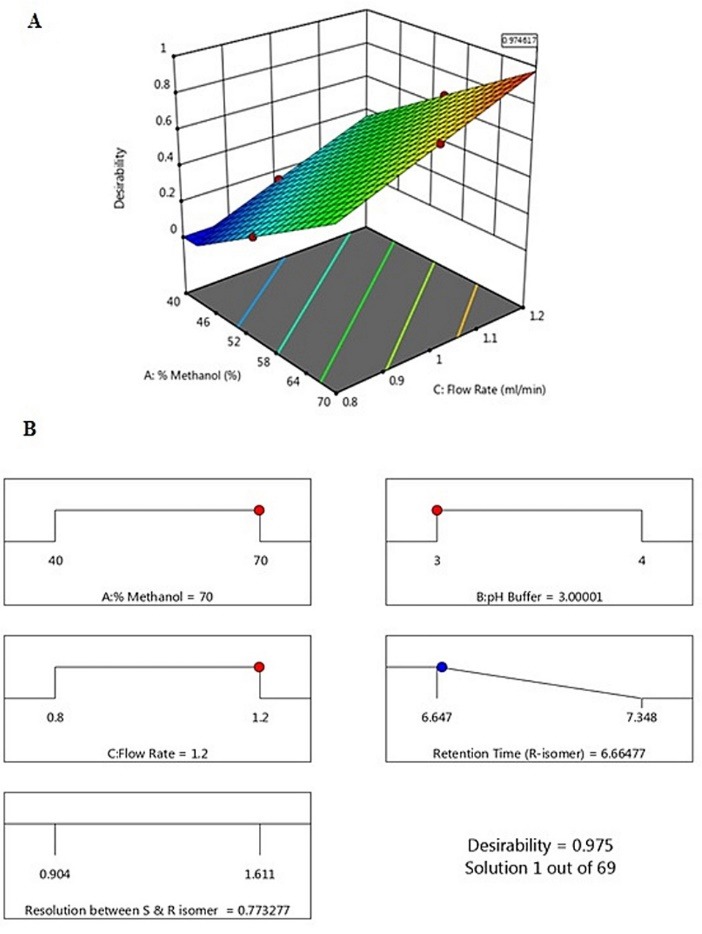
(A) The 3D surface plot of the overall desirability function Methanol% v/v is plotted against flow rate with pH of buffer held constant at 3.001. (B) Ramp view of the desirability function showing the most optimum chromatographic conditions for the ALO enantiomers quantification.

So, the ideal chromatographic condition as predicted by the statistical Box-Behnken model for the ALO enantiomer quantification is 70% Methanol, 30% 0.01% Formic acid buffer with pH 3 and flow rate 1.2 mL min^-1^ (Figure 3).

#### Optimization of the extraction process and selection of internal standard

The proposed method needed some modification for quantification of ALO enantiomers in plasma matrix due to limitations of the sensitivity of the method. The modifications involved the extraction of the ALO analytes and PIO from the rat plasma, evaporation of the solvent under low pressure and then recovery of ALO analytes and PIO in the least amount of methanol before analysis. PIO as an internal standard (Figure 1) gave sufficient response at 230 nm with a single nearly symmetric well-resolved peak from both the ALO enantiomers. C-18 extraction (SPE) cartridge was used for the extraction of the analytes from the plasma matrix. Only biomaterial was eluted while washing the plasma samples from the C-18 cartridge after its application.

**Figure 4:**
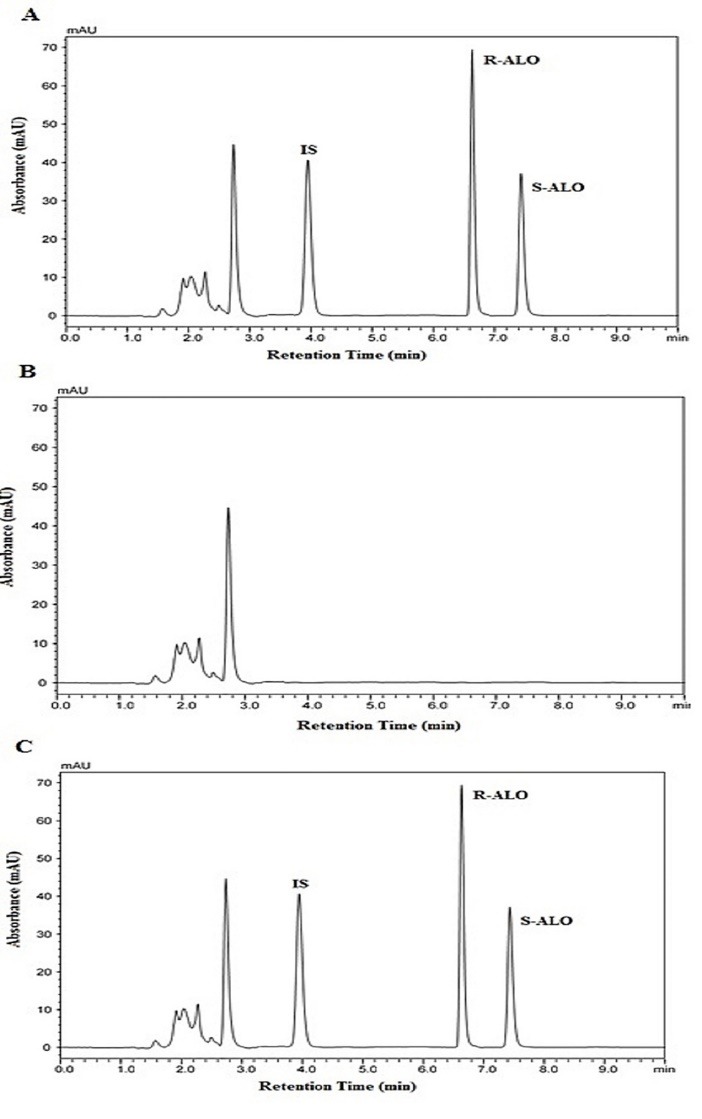
Chromatograms of (A) a mixture containing R-ALO (R, 7 µg/mL), S-ALO (S, 7 µg/mL) and PIO internal standard (IS, 7 µg/mL), (B) a blank plasma, and (C) a plasma sample spiked with R-ALO (R, 7 µg/mL), S-ALO (S, 7 µg/mL) and PIO (IS, 7 µg/mL), after SPE procedure using Phenomenex Lux Cellulose-2 chiral column and methanol:0.01% formic acid (70:30 v/v) mobile phase.

When the retained substances from the SPE cartridges were eluted with 2 mL of methanol and the eluate was distilled and the residue reconstituted in methanol, it was observed that the plasma components did not alter the results of analysis (Figure 4B). When the extraction process was used on the plasma matrix containing ALO and PIO analytes, peaks (Figure 4C) similar to the standard can be observed.

#### Validation

#### Specificity

The specificity was tested by applying developed HPLC method to tablets formulations and plasma samples. Pure peaks of the analytes (ALO and PIO) without any extra unwanted peaks were observed thus showing no interference from the tablet excipients or plasma components.

Also, the peak purities of R- ALO, S-ALO and PIO peaks were checked from the PDA data. The PDA also confirms selectivity of the methods by matching the peak purity spectra in the tablet as well as plasma matrix with the those in the standard solutions containing ALO enantiomers.

#### Linearity and calibration curve

In tablets and rat plasma matrix, 7 concentration levels were used to determine the linearity of the PDA response vs the analyte (R-ALO and S-ALO) concentrations. In case of formulation (tablet) the range was 10-70 μg mL^-1^ and in plasma it was 10-70 ng mL^-1^. The estimation of R and S-ALO in tablets is dependent on the area under curve for making the standard curves in the ranges given in Table 4. The lower limit of quantification (LLOQ) of analytes in plasma matrix is the concentration where the response to noise ratio was more than 10. The LLOQ in plasma matrix was found to be 1.2 ng. Statistical analysis^47^ data are summarized in Table 4.

**Table 4:** Linearity parameters used for the determination of R-ALO and S-ALO in plasma samples and tablets using the proposed HPLC method

**Parameter**	**Matrix**			
	**Plasma**		**Tablets**	
	**R-ALO**	**S-ALO**	**R-ALO**	**S-ALO**
Linearity range (µg/mL)	0.01-0.07	0.01-0.07	10-70	10-70
LOD (µg/mL)	0.0004	0.0003	0.20	0.21
LOQ (µg/mL)	0.0012	0.0012	0.61	0.62
Regression parameters^c^				
Slope (b)	0.9936	0.0614	1.008	1.008
Standard deviation of slope (S_b_)	0.0103	0.0083	0.002	0.001
Upper confidence limit of slope^d^	0.9973	0.0645	1.009	1.008
Lower confidence limit of slope^d^	0.9898	0.0584	1.008	1.008
Intercept(a)	0.0013	0.0078	0.087	0.102
Standard deviation of intercept (S_a_)	0.0004	0.0070	0.003	0.002
Upper confidence limit of intercept^d^	0.0014	0.0584	0.088	0.103
Lower confidence limit of intercept^d^	0.0012	0.0053	0.086	0.101
Standard deviation of residuals (S_y/x_)	0.0018	0.0019	1.875	1.276
Correlation coefficient (r)	0.9989	0.9978	0.9999	0.9998

^a^ Peak area ratio using PIO as an internal standard. ^b^Peak area. ^c^ Y=a+bC, where C is the concentration (mg mL^-1^) and Y is the peak area ratio or peak area. ^d^ 99% confidence limit.

#### Precision

The accuracy of the method was established by measuring the % recovery and % error (E_r_%) of a known amount of both R-ALO and S-ALO into plasma and tablet matrix. The accuracy was stated in terms of the percentage error (Er%) and the precision in RSD. The mean % recoveries obtained were 99.59 to 100.74 and 96.1 to 98.89 with mean percentage error below 0.94 and -1.11 in tablets and plasma, respectively for both the enantiomers.

T-test was applied for testing the accuracy of the method by comparing the within sample mean by its theoretical value. The t-values obtained were below theoretical values thus indicating the accuracy of the method (Table 5). The %RSD in both inter and intraday precision (Table 5) of the ALO analytes in tablets as well as human plasma were found to be below 1.44 and 3.83, respectively. So, all of these findings establish the acceptable levels of both precision and accuracy for both analytical^44^ and bioanalytical^45^ methods of analysis.

**Table 5:** Accuracy and precision of the determination of the ALO enantiomers in tablets and Rat plasma

**Matrix**	**Drug**	**Added conc.**	**Intraday**				**Interday**			
			**Mean recovery (%) ± SD**	**RSD (%)**	**Er%**	**t value** ^a^	**Mean recovery (%) ±SD**	**RSD (%)**	**Er%**	**t value** ^a^
Tablets (Conc in µg)	R-ALO	30	99.81±0.43	1.43	-0.19	0.72	100.87 ± 0.12	0.4	0.87	0.54
		40	100.6±0.46	1.15	0.6	1.43	100.48 ± 0.07	0.17	0.48	1.88
		50	100.68±0.28	0.57	0.68	1.32	100.69 ± 0.07	0.14	0.69	2.53
	S-ALO	30	100.94±0.13	0.41	0.94	2.05	100.7 ± 0.04	0.13	0.7	2.34
		40	100.76±0.15	0.38	0.76	2.38	99.59 ± 0.57	1.44	-0.41	1.98
		50	100.55±0.07	0.15	0.55	0.65	100.46 ± 0.04	0.08	0.46	0.34
Plasma (Conc. in ng)	R-ALO	15	97.64±0.02	1.31	-2.36	0.57	97.4 ± 0.03	2.02	-2.6	0.59
		25	97.54±0.08	3.31	-2.46	1.22	96.1 ± 0.06	2.31	-3.9	0.45
		40	96.1±0.14	3.54	-3.9	0.36	96.93 ± 0.06	1.65	-3.07	2.87
		55	97.36±0.08	1.41	-2.64	0.46	98.89 ± 0.06	1.05	-1.11	1.94
		65	97.56±0.09	1.35	-2.44	2.77	98.39 ± 0.05	0.71	-1.61	1.63
	S-ALO	15	97.12±0.03	2.25	-2.88	2.63	97.51 ± 0.03	1.77	-2.49	1.04
		25	98.56±0.08	3.25	-1.44	1.87	97.94 ± 0.04	1.55	-2.06	0.32
		40	95.94±0.09	2.25	-4.07	1.98	96.2 ± 0.1	2.63	-3.8	1.38
		55	98.31±0.09	1.7	-1.69	0.43	98.29 ± 0.07	1.25	-1.71	2.2
		65	98.52±0.25	3.83	-1.48	1.86	98.5 ± 0.08	1.25	-1.5	0.48

^a^The theoretical value for t-value (*P* = 0.01) is 4.60.

#### Robustness

For robustness study minor changes in the independent variables i.e. the wavelength, mobile phase composition (i.e. methanol%) and the flow rate (1±0.2 mL min^-1^) were done and the percent recovery of the enantiomers was observed. It was observed that small premeditated changes in the independent HPLC variables have negligible effects on the dependent variables (retention time and resolution between ALO analytes) thus establishing the robustness of the method.

#### Limit of detection and limit of quantification

Both limits of detection (LOD) and limits of quantification (LOQ) were obtained from ordinary least squares regression data. The calculated LOD and the LOQ data (Table 1) shows the sensitivity of the method and the ability to detect minute quantities of the ALO analytes in both the matrix (tablet and rat plasma).

#### Percentage recovery from matrix

Solid phase extraction (SPE) method using Oasis HLB 6 ml SPE cartridges from Waters Corporation was utilized for making the plasma samples with good sensitivity and recovery of the analytes. The percent recovery of all the quality control levels tested showed that the extraction efficacy of all the QC samples utilizing the extraction cartridges was good with negligible or no matrix effect.

#### Stability studies

Stability of analytes in plasma was evaluated in terms of freeze-thaw stability (three freeze-thaw cycles), benchtop stability (at room temperature for 8 hours), long-term stability and autosampler stability. In freeze-thaw stability, the chilled samples were thawed and maintained at room temperature. Benchtop stability was analyzed after keeping samples at room temperature for 24 hours. Long-term stability was analyzed after storing samples at -20°C for 20 days. In post-preparative stability study the samples were tested after keeping the samples for 24 hours at 4°C (post-preparative stability).

The stability studies performed (Table 6) showed that the ALO analytes in matrix can be stored at -20°C for 20 days and easily handled in normal lab conditions.

**Table 6:** Stability of ALO enantiomers in rat plasma

**Stability condition**	**Targeted conc. (ng/mL)**		**Mean Recovery (%)±SD**		**Er%**		**%RSD**	
	**R-ALO**	**S-ALO**	**R-ALO**	**S-ALO**	**R-ALO**	**S-ALO**	**R-ALO**	**S-ALO**
Short term stability	15	15	95.76±0.26	98.16±0.16	-4.24	1.78	1.78	1.58
	25	25	94.58±1.12	93.75±1.30	-5.42	4.76	4.76	5.43
	40	40	96.04±1.63	88.85±0.14	-3.97	4.25	4.25	7.54
	55	55	96.39±1.51	96.56±1.53	-3.61	2.85	2.85	3.07
	65	65	97.10±1.39	98.57±2.48	-2.90	2.20	2.20	1.35
Freeze thaw stability	15	15	95.11±0.19	101.11±0.08	-4.89	1.36	1.36	4.47
	25	25	97.92±0.34	98.65±0.64	-2.08	1.40	1.40	1.10
	40	40	97.54±0.70	96.96±1.27	-2.46	1.80	1.80	1.99
	55	55	97.95±0.98	98.69±2.13	-2.05	1.82	1.82	2.23
	65	65	98.44±0.91	98.36±2.93	-1.56	1.43	1.43	1.14
Long term stability	15	15	97.93±0.50	97.40±1.08	-2.07	3.37	3.37	2.57
	25	25	97.38±0.56	99.23±0.12	-2.62	2.30	2.30	4.60
	40	40	96.79±0.76	96.13±1.18	-3.22	1.96	1.96	2.59
	55	55	98.40±1.51	98.35±1.56	-1.60	2.80	2.80	2.98
	65	65	98.77±1.12	99.33±2.75	-1.23	1.74	1.74	2.02
Post preparative stability	15	15	95.11±0.66	96.49±1.79	-4.89	4.61	4.61	2.37
	25	25	94.97±0.91	98.17±0.78	-5.03	3.85	3.85	5.19
	40	40	95.93±0.80	95.83±1.10	-4.07	2.09	2.09	3.04
	55	55	98.31±0.59	99.31±2.55	-1.69	1.09	1.09	2.50
	65	65	98.23±0.67	98.72±3.15	-1.77	1.05	1.05	0.79

#### Analysis of pure ALO and tablets

The proposed HPLC-DAD method was applied to marketed ALO bulk forms and in formulations to determine the contents of both R and S-ALO enantiomers. The results showed that the concentrations of both were as per the literature available on the content of both enantiomers in the formulation (tablet) and raw material. The RSD (%) and Er (%) values for the assay showed the precise and accurate results of the developed method.

#### Application to pharmacokinetic study

The developed and validated method was used for the pharmacokinetic study of ALO analytes in 6 normal male rats after administering single oral dose of 3 mg kg^-1^. The quantities of both R-ALO and S-ALO in the rat blood plasma taken from the rats in the duration of one day were calculated.

**Figure 5:**
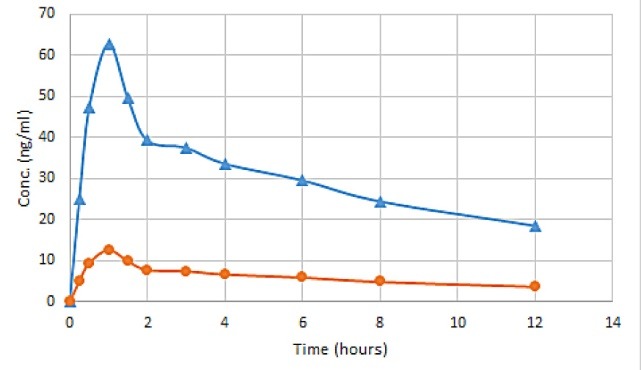
Mean plasma concentration-time profile of R-ALO (blue line) and S-ALO (orange line) following a single administration of a dose of 3 mg/kg of racemic alogliptin to 6 rats.

Figure 4 shows the illustrated chromatogram of the enantiomers and internal standard (PIO) in the rat plasma. The plasma concentration vs time proﬁle of R-ALO and S-ALO in rats is shown in Figure 5 . Plasma concentration-time data (Table 7) of each analyte were analyzed by the non-compartmental method using PK Solver software.^46^ The peak plasma concentration was reached by both R and S-ALO at approximately the equal time (in 1 hour) after administration of the oral ALO dose thus showing same absorption profile. The average *C*_max_ of *R*-ALO was greater than *S*-ALO by 4.96 times. The t_1/2_ of R-ALO and S-ALO was 9.175 and 9.169 hours respectively. The higher plasma concentration of the R-enantiomer as compared to the S-ALO may be because of greater rate of absorption of the R-ALO and/or extensive metabolism of the S-ALO than R-ALO. In liver the ALO is metabolized in two minor inactive metabolites (N demethylated metabolite and N-acetylated metabolite) which are excreted out through urinary pathway. Till now the stereoselective biotransformation of ALO and their effect on ALO quantities in plasma is not known in published literature.

**Table 7:** Assay results for the determination of ALO enantiomer in preparations

**Preparation**	**R-ALO**			**S-ALO**			**Ratio (R-ALO/S-ALO**
	Mean recovery (%)*	**Er%**	**CV (%)**	Mean recovery (%)*	**Er%**	**CV (%)**	
Raw material	98.73	-0.27	0.16	0.98	-2	0.91	≈99
Tablets	99.6	0.61	1.98	1.02	1.68	1.37	≈99

* Mean of five determinations.

## Conclusion

There may be significant variability between the enantiomers of chiral drugs in terms of their pharmacokinetics, efficacy, receptor affinity and toxicity. So, using a single enantiomorph of a racemic mixture may result in an improved therapeutic index, selective pharmacologic proﬁles and simple pharmacokinetics. Here an attempt has been made to develop a stereoselective RP-HPLC analytical method using Box-Behnken optimization method in the estimation of ALO enantiomorphs in formulations and in rat plasma. Derringer’s desirability function, a multi-criteria decision-making tool, was used to find the optimal chromatographic conditions. Methanol %, pH and the flow rate of the mobile phase were simultaneously optimized by applying useful tools of response surface design and Derringer’s desirability function. 70% methanol, 0.01% formic acid (30%) buffer with pH 3 and flow rate 1.2 mL min^-1^ was identified as the optimal condition by both mathematical and graphical methods. The developed optimized method was validated as per the ICH guidelines for analytical validation and tested for robustness, linearity, accuracy, and precision. Applicability of method was confirmed by analysis of ALO enantiomers in commercially available formulation and *in vivo* rat plasma samples.

The method proved to be selective, accurate, precise and robust for the determination of both ALO enantiomers. The validation data supported the fact that the assay method was specific, accurate, linear, precise, and robust for the estimation of R-ALO and S-ALO enantiomorphs. Therefore, this RP-HPLC method can be routinely used in quality control analysis in bulk as well as marketed formulations and pharmacokinetics study in rats.

While the active R-ALO in rat plasma was considerably more as compared to inactive S-ALO after single dose of racemic ALO in physically normal rats still S isomer was found in significant proportions in the rat plasma. It may be assumed that the enantiomer R-ALO would be a better drug in comparison to racemic ALO. Further studies are needed to be conducted to assess the side effects of inactive S-isomer in significant proportion in the blood plasma.

## Ethical Issues

The study was approved by Institutional Ethical Committee, Delhi Institute of Pharmaceutical Sciences and Research, New Delhi, India.

## Conflict of Interest

None.

## Acknowledgments

We are thankful to Sun Pharmaceutical Industries Limited (Gurugram, Haryana, India) for providing the gift sample of ALO enantiomers. Also, we are grateful to Delhi Institute of Pharmaceutical Sciences and Research, University of Delhi for providing the infrastructure. We are thankful to the University Grants Commission, New Delhi for providing the fund for the research work.
